# Does the different function of the lead vs. trail foot lead to functional asymmetry in balance performance of sub-elite youth cross-country mountain bikers? A pilot study

**DOI:** 10.3389/fspor.2026.1944180

**Published:** 2026-09-30

**Authors:** Sam Limpach, Matthias Lauer, Moritz Schreiber, Katharina Borgmann, Thomas Muehlbauer, Stefan Panzer

**Affiliations:** 1 Institute of Sport Science, Saarland University, Saarbrücken, Germany; 2 Saarländischer Radfahrer-Bund, Saarbrücken, Germany; 3 Division of Movement and Training Sciences/Biomechanics of Sport, University of Duisburg-Essen, Essen, Germany; 4 Institute of Biomechanics and Orthopaedics, German Sport University Cologne, Cologne, Germany; 5 Department of Health and Kinesiology, Texas A&M University, College Station, TX, United States

**Keywords:** functional asymmetry, mountain biking, postural control, time to stabilization, Y-balance test-lower quarter

## Abstract

**Background:**

During descents, cornering, or coasting over obstacles, cross-country mountain bikers use an isometric split stance with horizontally aligned pedals, establishing a distinct “lead” and “trail” leg. This pilot study investigated whether the different functions of the lead compared to the trail foot are associated with inter-limb functional asymmetry (FA) in proactive and reactive balance performance.

**Methods:**

Twelve sub-elite youth cross-country mountain bikers (10 males, age: 13.5 ± 1.7 years, training history: 4.7 years) were assessed. Proactive balance was evaluated using the Y-Balance Test-Lower Quarter (YBT-LQ). Reactive balance was assessed via Time to Stabilization (TTS) during single-leg drop landings. Statistical analyses utilized Kruskal–Wallis H-tests and Wilcoxon signed-rank tests to compare performance differences between the lead and trail leg.

**Results:**

No significant differences between the lead and trail leg were observed across any reach directions or the composite score of the YBT-LQ. Contrarily, TTS revealed a significant difference (*Z* = 2.59, *p* = .010, *r* = .75) for the mediolateral plane, with the lead leg having a faster TTS.

**Discussion:**

Chronic participation in mountain biking was associated with an FA in measures of reactive balance specifically isolated to the ML plane. Since off-road descents present unpredictable, high-speed perturbations, the rider must rely heavily on a mediolateral “hip strategy.” The superior reactive stabilization of the lead leg suggests that this specific FA could serve as a sport-specific performance indicator.

## 1 Introduction

It is well established that the human neuromusculoskeletal system is highly adaptable to its environmental constraints (1). Consequently, chronic exposure to high-level, sport-specific demands drives significant structural and functional adaptations (2). Postural control is no exception to this phenomenon. Enhancements in balance are highly sport- and environment-specific, driven by the optimized integration of visual, vestibular, and proprioceptive information (3, 4).

A prominent example of this sensory optimization is presented by Lion et al. (5) across different cycling disciplines. The authors demonstrated that road cyclists rely predominantly on visual input for postural control, whereas mountain bikers depend significantly more on proprioceptive information. This difference in sensory reliance emerges primarily from the different environments in which road cyclists and mountain bikers operate. Mountain bikers navigate through rough terrain with changes in surface conditions, which places a higher emphasis on proprioceptive input, as their neuromuscular system must quickly adapt to sudden physical perturbations that could not have been anticipated solely through visual information.

Navigating this unpredictable terrain requires a highly specialized riding posture. During descents, cornering, or coasting over obstacles, mountain bikers adopt a neutral pedal position where the cranks are held horizontally (6). This inherently forces the rider into an isometric split stance, establishing a distinct “lead” leg and “trail” leg (7, 8). The lead leg, positioned toward the front wheel, is typically held slightly higher and is primarily responsible for guiding the rider's body position and balance through turns. Conversely, the trail leg is positioned toward the rear wheel, carrying more of the rider's body weight to provide foundational stability and control over rough terrain (7, 8). Importantly, based on current coaching manuals and established coaching pedagogy, riders consistently adopt a single, preferred “lead” and “trail” leg during descents rather than alternating stances (6, 9).

The chronic utilization of such specialized stances may lead to the development of functional asymmetry (FA) in balance capabilities (3, 4). FA is defined as an asymmetrical capability in postural control between the limbs. This phenomenon is well-documented in sports, such as soccer, where the accumulation of training years and competitive level achieved, combined with the distinct roles of a preferred kicking leg and a stabilizing stance leg, creates measurable inter-limb asymmetries in postural control (10–12). While small degrees of FA can be beneficial for specific athletic actions (13), side differences in single-leg drop jump height of 10%–15% or an anterior reach difference of more than 4 cm in the Y-Balance Test-Lower Quarter (YBT-LQ) are accompanied with a decrease in athletic performance and an increase in the risk of non-contact lower limb injuries (14–16).

However, empirical support for FA in mountain biking is limited. Since mountain biking requires both the proactive shifting of body weight to navigate turns and the reactive absorption of sudden terrain impacts, a comprehensive evaluation of FA requires assessing both proactive and reactive measures of balance. Therefore, the present study was designed to investigate whether sub-elite young mountain bikers exhibit FA during a proactive (YBT-LQ) and a reactive [Time to Stabilization (TTS) during single-leg drop landings] balance task. The YBT-LQ was chosen because it requires athletes to proactively shift their center of mass to its limits of stability across multiplanar directions while getting into a split stance, mirroring the weight-shifting mechanics required during cornering (17, 18). Conversely, TTS during single-leg drop landings was selected as it mirrors the impact absorption phase following a drop or jump on a mountain bike (19). Additionally, the reactive balance task provides an opportunity to investigate the use of different postural control strategies, the ‘hip strategy’, which is presented by movements in the mediolateral (ML) plane, and the ‘ankle strategy’, which is mainly shown by movements in the anteroposterior (AP) plane (20).

According to Paillard (3), it is hypothesized that the chronic utilization of such specialized stances leads to the development of FA. Further, based on the distinct biomechanical roles adopted during the neutral riding stance, we hypothesized that, because the lead leg is primarily responsible for guiding body position and balancing through turns, proactive and reactive FA will be weighted toward better balance control for the lead leg than for the trail leg. As movement techniques must be used to respond to the demands of the terrain, which primarily involve rapid changes in direction, it was hypothesized that this FA would be more pronounced in the ML plane.

## 2 Materials and methods

### 2.1 Participants

For this cross-sectional study, twelve youth cross-country mountain bikers, classified as Tier 3 (highly trained/national level) according to McKay et al. (21), were recruited. All participants were active members of the Saarland regional representative squad (*Landeskader Saarland*) (yielding a cumulative record of 11 national victories, 22 podium finishes, and 63 top-ten placements during the 2024-2025 season). Given the population's elite nature, convenience sampling was used to recruit the entire available squad. The sample consisted of ten males and two females. A comprehensive overview of the participants’ characteristics is depicted in Table 1.

**Table 1:** Characteristics of the participants.

Characteristic	Value
Age (years), *M* (±*SD*)	13.5 (±1.73)
Sex (male/female), *n*	10/ 2
Height (cm), *M* (±*SD*)	170.32 (±6.02)
Mass (kg), *M* (±*SD*)	57.49 (±1.79)
Training experience (years), *M* (±*SD*)	4.71 (±1.98)
Training volume (min/week), M (±*SD*)	698.75 (±197.30)
Maturity offset stage, *n*^a^	−1 (*n* = 6); 0 (*n* = 4); +1 (*n* = 2)

a Based on the research of Moore et al. (22).

Regarding their preferred downhill stance, four riders preferred the right foot as the lead leg, while eight riders preferred the left leg. Participants did not have any history of musculoskeletal, neurological, or orthopedic pathologies within the preceding six months. Before enrollment, the study procedures were explained to both the athletes and their legal guardians. Written informed consent was obtained from the legal guardians, and assent was provided by the participants. The study was conducted in accordance with the Declaration of Helsinki (World Medical Association, 2013).

### 2.2 Experimental procedure

Upon arrival at the biomechanics laboratory, anthropometric data (body height, body mass) and training experience (years of mountain biking, years in the regional squad) were collected. Additionally, the preferred leg positioning during downhill mountain biking was asked. The preferred forward leg was defined as the lead leg, while the rear leg was designated as the trail leg for all subsequent analyses.

Participants received standardized verbal and visual instructions about the assessment protocols (YBT-LQ and single-leg drop landings). Then, a standardized 10-minute warm-up was performed, including static (e.g., unipedal stance), dynamic (e.g., beam walking), reactive (e.g., jump landings), and proactive (e.g., maximal leaning) balance exercises. The order of the two assessments was randomized for each participant to reduce sequence effects. Before recording data, a familiarization period was provided for each test, during which any execution errors were corrected by trained investigators. All testing sessions were conducted by the same two investigators (sport scientists with a Master's degree) to ensure consistency.

### 2.3 Assessment of anthropometric variables

Body height was measured without shoes to the nearest 0.5 cm using a stadiometer (seca 217, Basel, Switzerland), and body weight was measured to the nearest 0.1 kg using an electronic scale (seca 803, Basel, Switzerland). To normalize the YBT-LQ reach distances, anatomical leg length was measured for both limbs. Participants lay in a supine position, and leg length was defined as the distance from the anterior superior iliac spine (ASIS) to the most distal point of the medial malleolus, measured to the nearest 0.2 cm with a measuring tape (18).

### 2.4 Assessment of balance performance

Reactive balance was measured using a single-leg drop-landing task. Participants stood on a box (height: 30 cm) in a single-leg stance with their hands placed on the iliac crests. They were instructed to “step off” the box with the non-weight-bearing leg, avoiding any upward vertical propulsion or jumping motion, and land on an unstable foam pad (Airex Balance Pad, Airex AG, Sins, Switzerland) placed centrally on top of a force plate (Kistler, 9260AA, Winterthur, Switzerland). Upon landing, participants were asked to stabilize as quickly as possible and maintain the single-leg stance for 15 s. Following two familiarization trials per leg, three valid data-collection trials were recorded for both the lead and trail leg. The primary outcome variable extracted from the drop landings was the ground reaction forces (GRF), which were subsequently used to calculate the time to stabilization (TTS), measured in seconds. The testing order was randomized. A trial was deemed invalid and repeated if: (1) the hands were removed from the iliac crests, (2) the participant jumped rather than dropped, (3) the non-standing leg touched the ground or the contralateral leg before 15 s passed by. Trials were repeated until three valid attempts per leg were obtained. Validity and reliability of this protocol have been established in previous studies (19, 23).

Proactive balance was assessed using the YBT-LQ kit (Functional Movement Systems, Chatham, VA, USA). Participants stood unilaterally on the central platform with their hands on the iliac crests. With the contralateral limb, they were instructed to push the reach indicator blocks as far as possible in the anterior (ANT), posteromedial (PM), and posterolateral (PL) directions. A standardized protocol was followed, consisting of three familiarization trials and three data-collection trials per reach direction for each leg. The sequence of legs was randomized. A trial was considered invalid and repeated if the participant: (1) failed to maintain unilateral stance (e.g., touched the floor with the reaching foot), (2) placed weight on the reaching indicator block, (3) kicked the indicator block rather than pushing it, (4) removed hands from the iliac crests, or (5) lifted the heel of the stance leg from the central platform. The maximum reach distance from the three valid trials was recorded for further analysis. The YBT-LQ has been demonstrated to be valid and reliable (15, 18, 24).

### 2.5 Data processing and analyses

Regarding the TTS, ground reaction force (GRF) data were acquired at 1,000 Hz. Post-acquisition, data was low-pass filtered with a cut-off frequency of 10 Hz using a second-order Butterworth filter. All signal processing was conducted using a custom script written in MATLAB R2023a (The MathWorks, Natick, MA, USA). The TTS was calculated using the sequential average (SA) method, as this algorithm has demonstrated superior reliability for both AP and ML planes (19). Initial contact was defined as the first sample at which the vertical GRF exceeded 10 N. Starting from initial contact, the SA was calculated separately for the vertical, AP, and ML GRF components. At each sample, the SA represented the cumulative mean of all GRF values from initial contact up to the respective sample. For each GRF component separately, the stabilization range was defined as the mean of the entire analyzed post-landing time series ±0.25 standard deviations of the respective signal. TTS was defined as the first time point at which the SA entered this stabilization range and remained within it for the remainder of the analyzed period. The same SA procedure was applied separately to the vertical, AP, and ML components. Additionally, a TTS composite score (CS) was calculated from the AP and ML TTS values according to the following equation:

$$Composite\;Score_{TTS}\;\left(s\right)=\sqrt{{\mathrm{TTS}}_{\mathrm{AP}}^{2}+{\mathrm{TTS}}_{\mathrm{ML}}^{2}}$$

For the YBT-LQ, the maximum reach distance (cm) from the three valid trials per reach direction was used for analysis. Reach distances were normalized to anatomical leg length using the following equation:

$$Normalized\;Reach\;\left(\%\right)=\frac{\mathrm{Maximal}\;\mathrm{Reach}\;\mathrm{Distance}\;\left(\mathrm{cm}\right)}{\mathrm{Leg}\;\mathrm{Length}\;\left(\mathrm{cm}\right)}x100$$

Additionally, a composite score (CS) was calculated to represent overall proactive balance performance:

$$Composite\;Score\;\left(\%\right)=\frac{\mathrm{Sum}\;\mathrm{of}\;3\;\mathrm{Maximal}\;\mathrm{Reach}\;\mathrm{Distances}\;\left(\mathrm{cm}\right)}{3x\mathrm{Leg}\;\mathrm{Length}\;\left(\mathrm{cm}\right)}x100$$

To quantify FA between the downhill stance positions, a limb symmetry index (LSI) was calculated for both TTS and YBT-LQ scores, based on the formula proposed by Bishop et al. (25):

$$LSI\;\left(\%\right)=\left(1-\frac{\mathrm{Result}\;\mathrm{Trail}\;\mathrm{Leg}}{\mathrm{Result}\;\mathrm{Lead}\;\mathrm{Leg}}\right)x100$$

An LSI value deviating from zero indicates asymmetry, with positive values indicating better performance of the trail leg regarding the YBT-LQ data. Regarding the single-leg drop-landings, higher values indicate better performance for the lead leg. An asymmetry magnitude greater than 10% was considered clinically significant (25).

### 2.6 Statistical analysis

Descriptive statistics are presented using box-and-whisker plots showing the smallest observation (sample minimum), the lower quartile (25th percentile), the median (50th percentile), the upper quartile (75th percentile), and the largest observation (sample maximum). Normality of the data was assessed using the Shapiro–Wilk test for all directional and composite scores of both the YBT-LQ (ANT, PM, PL, and CS) and the TTS during single-leg drop landings (AP, ML, vertical, and CS).

Since several sub-scores, specifically for the YBT-LQ the PM: (*p* = .04), the PL: (*p* = .01), and for the TTS the ML (*p* < .001), significantly violated the assumption of normal distribution, non-parametric statistical procedures were uniformly applied for all subsequent analyses. Initially, a Kruskal–Wallis H-test was conducted to evaluate potential global differences across the various directional sub-scores of the YBT-LQ and the TTS. Following this, Wilcoxon signed-rank tests (*Z*-value) were used to assess any significant differences between the lead and trail leg. For the Wilcoxon signed-rank tests, Cohen's *r* was calculated and interpreted as small (*r* < .1), weak (*r* < .3), moderate (*r* < .5), or large (*r* ≥ .5). For the Kruskal–Wallis H-tests, eta-squared (*η*^2^) was calculated and interpreted as small (.01-.05), moderate (.06-.13), or large (>.14). To ensure the robustness of the findings, a sensitivity analysis was pre-defined to be conducted for any variable presenting extreme outliers (e.g., data points falling outside 3 interquartile ranges), evaluating whether significance and effect sizes were strictly driven by single data points.

The alpha level for statistical significance was set *a priori* at *α*<.05. All statistical analyses were performed using a custom-written script in R version 4.5.2 (2025-10-31), R Core Team, Vienna, Austria) within the RStudio environment (Posit Software, PBC, Boston, MA, USA).

## 3 Results

### 3.1 Proactive balance (YBT-LQ)

Table 2 contains descriptive data of the median and IQR for all the sub scores of the YBT-LQ. The Kruskal–Wallis H-test revealed no significant differences in the LSI across the different reaching directions [*χ*^2^(2) = 0.52, *p* = .773, *η*^2^ = .00] (see Figure 1). Furthermore, subsequent Wilcoxon signed-rank tests indicated no significant LSI-values, neither for the ANT [3.57 (11.62); *Z* = 1.60, *p* = .11, *r* = .41], PM [0.43 (3.06); *Z* = .78, *p* = .43, *r* = .21], and PL [4.44 (2.48); *Z* = 1.43, *p* = .15, *r* = .40] reach directions, nor for the overall CS [2.54 (4.52); *Z* = 1.22, *p* = .22, *r* = .34] (see Figure 2).

**Table 2:** Descriptive statistics and interlimb symmetry for the proactive Y-balance test.

Parameter	Lead leg (%LL)	Trail leg (%LL)	LSI (%)
Anterior	71.11 [4.16]	70.22 [4.21]	3.57 [11.62]
Posteromedial	108.89 [10.64]	107.07 [7.41]	0.43 [3.06]
Posterolateral	109.89 [12.01]	110.15 [10.46]	4.44 [2.48]
Composite score	96.30 [9.99]	96.31 [7.00]	2.54 [4.52]

LSI, interlimb symmetry index; %LL, percentage of leg length. All data are presented as median [interquartile range].

**Figure 1:**
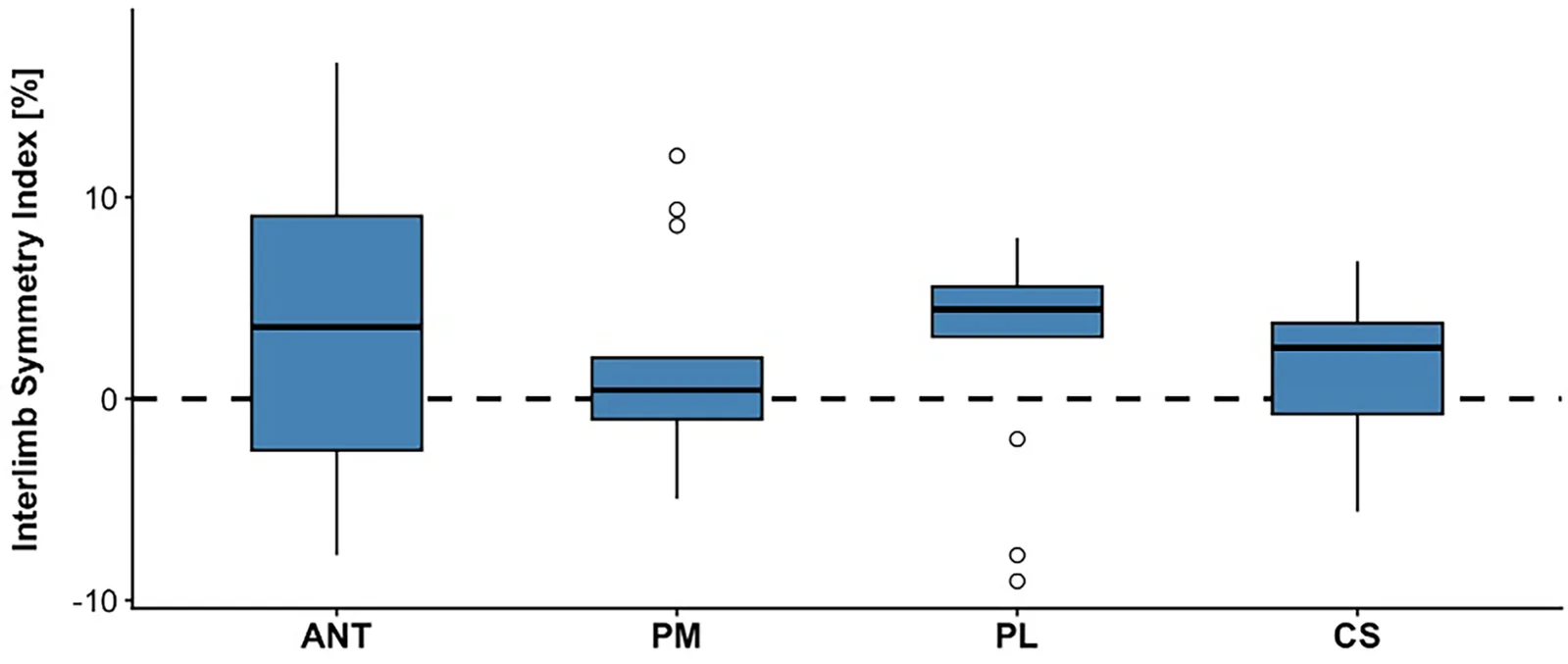
Box-and-whisker plots for the limb symmetry index across all reach directions and the composite score of the Y-balance test-lower quarter. A positive LSI indicates a superior performance of the lead leg, whereas a negative LSI indicates a superior performance of the trail leg.

**Figure 2:**
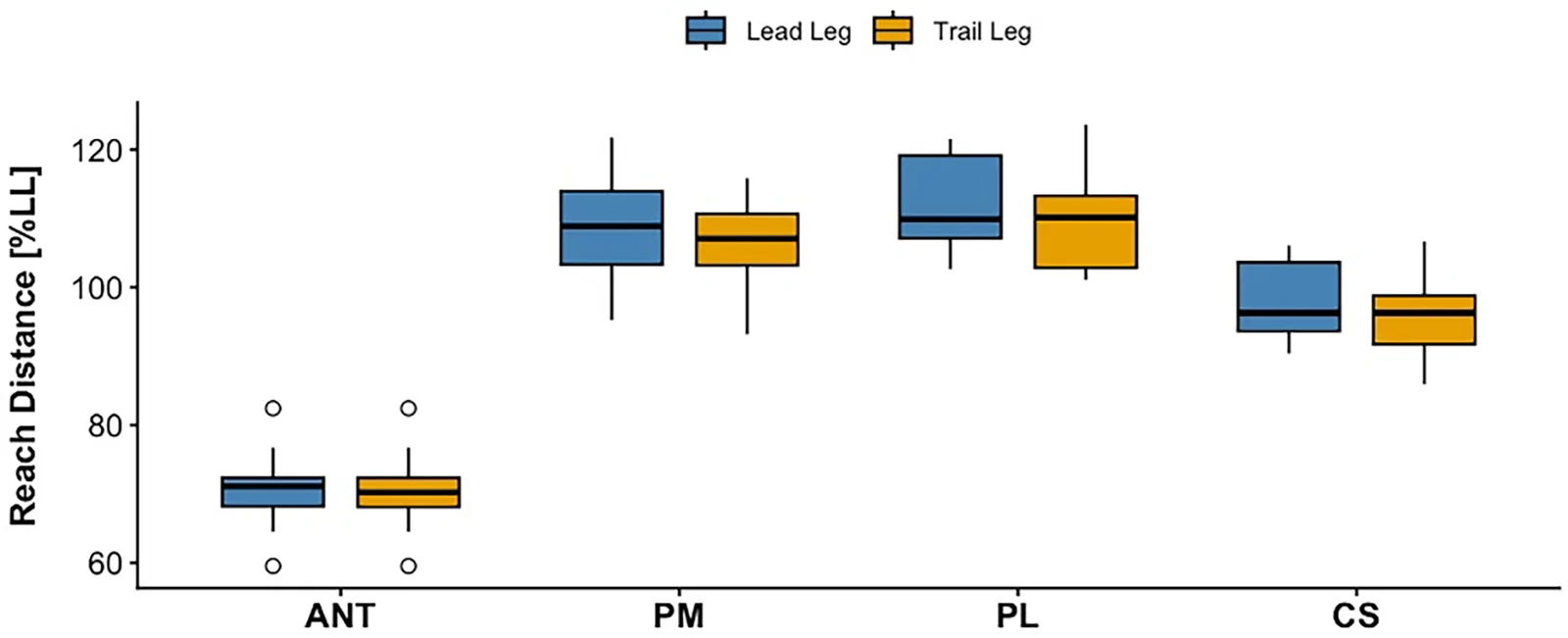
Box-and-whisker plots comparing the reach distances for the sub-scores of the Y-balance test-lower quarter between the lead and trail leg. LL, leg length.

### 3.2 Reactive balance (drop landings)

Table 3 contains descriptive data of the median and IQR for all the sub scores of the TTS. An initial Kruskal–Wallis H-test comparing the LSI across the different balancing directions revealed a significant difference with a large effect size [χ^2^(2) = 7.261, *p* = .026, *η*^2^ = .159] (see Figure 3). Subsequent Wilcoxon signed-rank tests indicated a significant difference in the ML plane. The lead leg demonstrated a significantly faster stabilization time than the trail leg [30.47 (22.32); *Z* = 2.59, *p* = .010, *r* = .75]. Because an extreme outlier was identified in the mediolateral TTS data of the lead leg, a sensitivity analysis was conducted. After excluding the outlier (*n* = 11), the Wilcoxon signed-rank test confirmed that the significant difference between the lead and trail leg remained robust (Z = 2.40, *p* = .016, *r* = .72). Therefore, the observed asymmetry in the ML plane was not solely driven by this single extreme value. Additionally, to evaluate individual consistency, examination of the paired data revealed that ten out of twelve participants exhibited faster mediolateral stabilization times on their preferred lead leg, indicating a consistent directional trend across the cohort. No significant LSI-values were detected for the vertical [6.29 (43.42); *Z* = .24, *p* = .81, *r* = .07], AP [3.27 (5.04); *Z* = 1.96 *p* = .05, *r* = .56], and CS [−5.17 (13.03); *Z* = 1.57, *p* = .12, *r* = .45] (see Figure 4).

**Table 3:** Descriptive statistics and interlimb symmetry for reactive drop landings.

Parameter	Lead leg (s)	Trail leg (s)	LSI (%)
Vertical	2.45 [0.53]	2.31 [1.57]	6.29 [43.42]
Anteroposterior	5.12 [0.12]	4.92 [0.37]	0.43 [3.06]
Mediolateral	2.37 [1.66]	3.13 [1.58]	−30.47 [22.34]
Composite score	5.71 [0.82]	5.93 [1.01]	−5.17 [13.03]

LSI, interlimb symmetry index. All data are presented as median [interquartile range].

**Figure 3:**
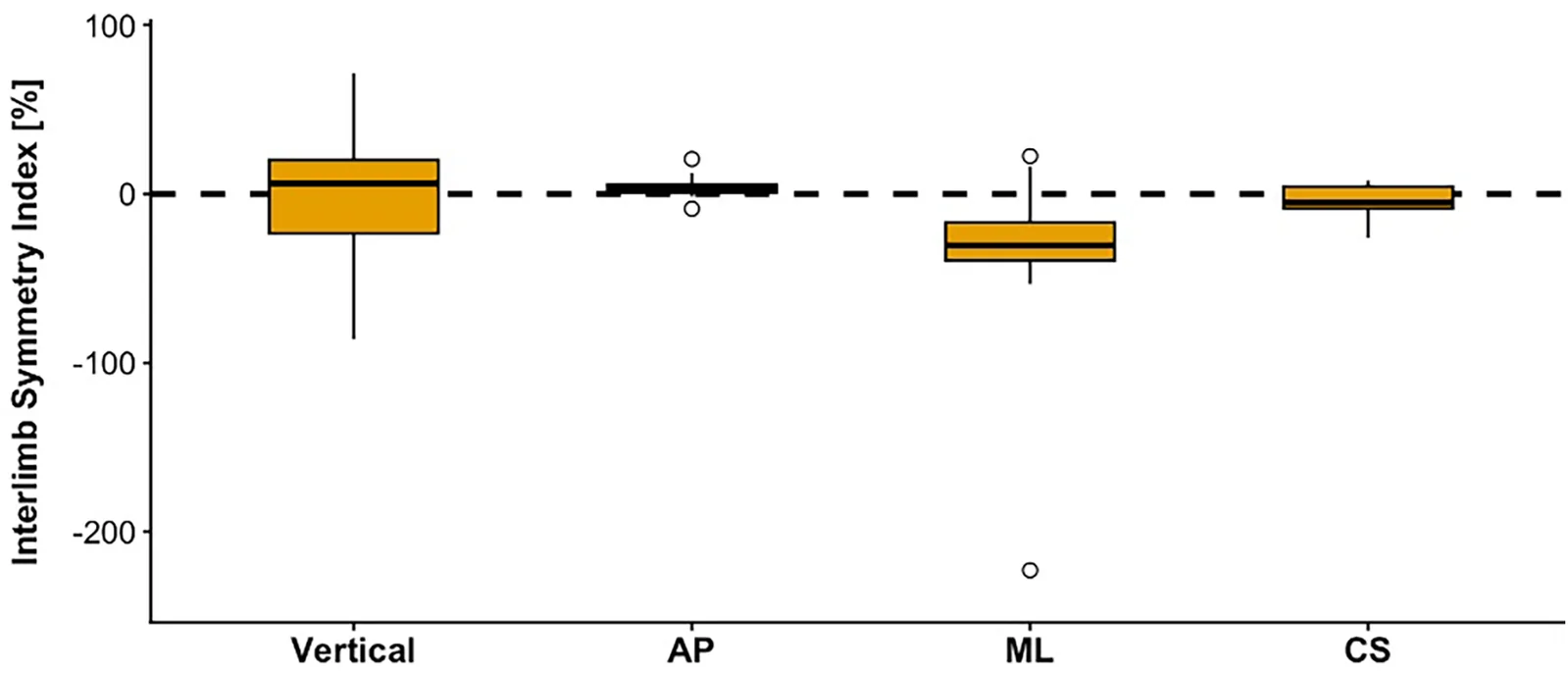
Box-and-whisker plots for the limb symmetry index across all force planes and the composite score of the single-leg drop landing test. A positive LSI indicates a superior performance of the trail leg, whereas a negative LSI indicates a superior performance of the lead leg.

**Figure 4:**
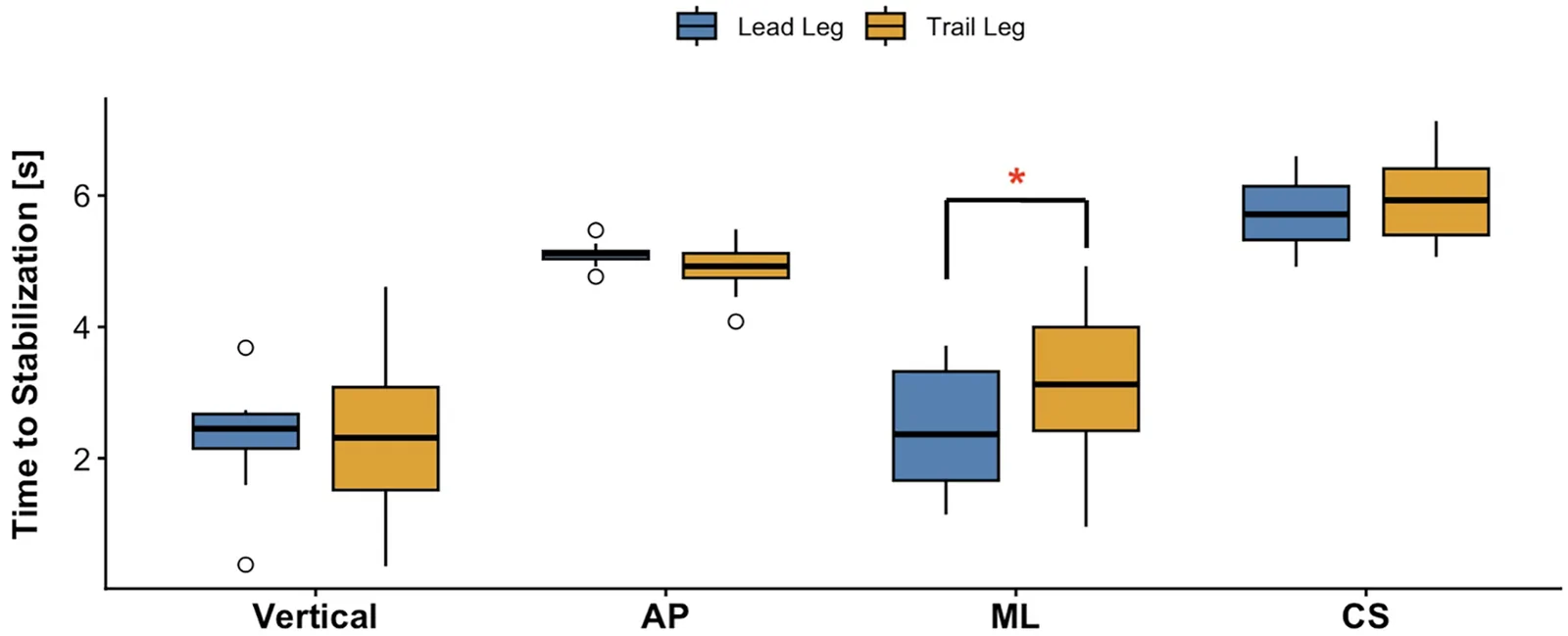
Box-and-whisker plots comparing the time to stabilization for the sub-scores of the single-leg drop landing test between the lead and trail leg. *Indicates a significant inter-limb asymmetry (*p* < .05).

## 4 Discussion

The present pilot study aimed to investigate whether sub-elite young mountain bikers have a FA in lower-limb balance capabilities between the lead and trail leg due to the chronic, asymmetrical postural demands of the neutral pedal split stance. To obtain a comprehensive profile of these balance capabilities, two sub-categories of postural control were assessed: proactive balance (reach distances in the YBT-LQ) and reactive balance (TTS during single-leg drop landings). The primary finding of this investigation is that FA emerged during the reactive balance task, with the lead leg demonstrating a significantly faster stabilization time exclusively in the ML plane following the single-leg drop landings. While no significant functional asymmetry was observed during the proactive balance task (YBT-LQ), these results must be interpreted with caution. Given the small sample size of this pilot study, we cannot definitively conclude that proactive FA does not exist in this population. Rather, the study may have been underpowered to detect it, potentially resulting in a Type II error.

Proactive balance results from the anticipatory adjustment of postural control before performing a potentially destabilizing movement. Visual and proprioceptive input is analyzed to evaluate potential risks and proactively activate muscles to keep an upright stance (26). We hypothesized that the chronic split stance in mountain biking would induce a FA between the lead and trail leg. However, the data did not reveal significant side-to-side differences. Specifically, across all reach directions and the CS, no significant differences in measures of proactive balance performance were observed. This absence of proactive FA may be attributed to the multifactorial nature of the YBT-LQ. While the split stance places different proprioceptive loads on the limbs, performance is also heavily influenced by lower-extremity strength, neuromuscular coordination, movement strategy and joint range of motion (17). It is plausible that the physical demands of riding distribute strength and mobility adaptations relatively symmetrically, overshadowing any purely proprioceptive asymmetries.

In contrast to the findings for proactive balance, the assessment of reactive balance, defined as the rapid, automatic responses required to restore balance following unexpected external disturbances (27), revealed a significant FA between the lead and trail leg specifically restricted to the ML plane. During the single-leg drop landings, the lead leg demonstrated a significantly faster TTS exclusively in the ML plane. This finding provides partial support for the second hypothesis. The associated effect size is large, indicating that the preferred lead leg possesses a significantly faster capacity to stabilize in the ML plane compared to the preferred trail leg.

This specific adaptation could be explained biomechanically by the magnitude of perturbations encountered during mountain biking and/or by the feet being mechanically fixed to the pedals via the click system. According to Winter et al. (20) human postural control relies on two distinct mechanisms, the ‘ankle strategy,’ which governs balance in the AP plane, and the ‘hip strategy,’ which dictates stabilization in the ML plane. While postural control on a mountain bike is achieved through a combination of ankle and hip strategies, their deployment depends heavily on the degree of imbalance (28). The ankle strategy is typically sufficient for small, subtle adjustments to maintain stability in a neutral position. However, the hip strategy, involving pelvic flexion and rapid lateral shifting of body weight, is strictly required to cope with larger disturbances and steep, technically demanding off-road terrain including change of direction and navigating rough and winding terrain (28).

Furthermore, while modern mountain bikes utilize advanced suspension forks, rear shocks, and long wheelbases that effectively absorb vertical and fore-aft (AP) impacts, bicycles cannot mechanically attenuate ML perturbations. The rider's lower limbs must manage these lateral forces entirely. Consequently, the lead leg is subjected to chronic stabilization demands in the ML plane to counteract lateral shifts, providing a possible biomechanical rationale for why functional asymmetry was specific to the mediolateral direction.

In accordance with the assumption that the lead leg supports body position and balance (7, 8), this limb naturally endures a higher load when executing the hip strategy during downhill descents. While the rider is in the neutral split stance, mountain biking presents a high volume of rapid balance disturbances that cannot be proactively foreseen, especially when riding at a higher speed (6). Consequently, the lead leg must continuously manage these unpredictable perturbations, developing the FA observed in the present study.

The significantly faster ML stabilization time of the lead leg suggests that, unlike FA in balance in field sports, this inter-limb asymmetry may not indicate an increased injury risk or diminished athletic output.

### 4.1 Future directions and limitations

As a pilot study, several limitations must be acknowledged. Foremost is the small sample size (*n* = 12) and the use of less powerful non-parametric statistical procedures, meaning the absence of significant differences in the proactive YBT-LQ may be the result of a Type II error due to insufficient statistical power. Second, with a mean age of 13.5 years, the youth athletes in this cohort are navigating peak height velocity (PHV). Rapid physical maturation and the resulting “adolescent awkwardness” are known to temporarily disrupt proprioception and motor control (29), which adds inherent noise to balance assessments. Furthermore, the uneven sex distribution (10 males, 2 females) limits generalizability, as differences in the Q-angle and center of mass could influence ML stabilization times. Because this is a cross-sectional study lacking a non-biking control group, causality cannot be definitively established. We cannot rule out that athletes with a better ML reactive balance in one limb simply select that limb as their lead leg. Finally, external confounding variables, such as strength training, school sports, and other daily activities, were not controlled for. Future research should include larger, adult cohorts with longer chronic exposure to the asymmetric stance to verify these associative findings.

## 5 Conclusion

This study demonstrated that chronic participation in cross-country mountain biking was associated by an FA in reactive balance (i.e., in the ML plane of the lead leg). While this finding positions cross-country mountain biking alongside sports with asymmetrical balance workloads like soccer, their consequences are still unclear, as it could, opposed to field sports, be a performance indicator. Rather than strictly predisposing athletes to injury, the increased reactive stabilization of the guiding lead leg could represent a vital, sport-specific performance adaptation necessary for high-level competition.

Thus, future research should investigate whether this FA is a performance enhancer or if, just like in field sports, it diminishes performance and increases injury rates. Using EMG and IMU based analyses could also further deepen the knowledge on possible equal load distribution of the ‘lead’ and ‘trail’ leg of proprioceptive balance. Additionally, identifying precise thresholds for these beneficial or detrimental effects is imperative. Establishing whether higher degrees of FA in the ML plane correlate with faster downhill race times will enable coaches and sports scientists to better monitor athletes and optimize unilateral training interventions without blindly attempting to correct functional, beneficial asymmetries.

## Data Availability

The data generated and analysed during the present study are not publicly available due to ethical restrictions but are available from the corresponding author upon reasonable request.
